# Identification of small extracellular vesicle protein biomarkers for pediatric Ewing Sarcoma

**DOI:** 10.3389/fmolb.2023.1138594

**Published:** 2023-04-13

**Authors:** Soumya M. Turaga, Mihaela E. Sardiu, Vikalp Vishwakarma, Amrita Mitra, Leonidas E. Bantis, Rashna Madan, Michael L. Merchant, Jon B. Klein, Glenson Samuel, Andrew K. Godwin

**Affiliations:** ^**1**^ Department of Pathology and Laboratory Medicine, The University of Kansas Medical Center, Kansas City, KS, United States; ^2^ Department of Biostatistics and Data Science, The University of Kansas Medical Center, Kansas City, KS, United States; ^3^ Kansas Institute for Precision Medicine, The University of Kansas Medical Center, Kansas City, KS, United States; ^4^ University of Kansas Cancer Center, Kansas City, KS, United States; ^5^ Clinical Proteomics Laboratory, Department of Medicine, University of Louisville, Louisville, KY, United States; ^6^ Robley Rex Veterans Administration Medical Center, Louisville, KY, United States; ^7^ Division of Pediatric Hematology Oncology and Bone Marrow Transplantation, Children’s Mercy-Kansas City, Kansas City, MO, United States

**Keywords:** ewing sarcoma, proteomics, mass-spectrometry, small extracellular vesicles, exosomes, sEV-associated protein biomarkers, liquid biopsy, EWS-ETS fusions

## Abstract

Ewing Sarcoma (EWS) is the second most common osseous malignancy in children and young adults after osteosarcoma, while it is the fifth common osseous malignancy within adult age population. The clinical presentation of EWS is quite often non-specific, with the most common symptoms at presentation consisting of pain, swelling or general discomfort. The dearth of clinically relevant diagnostic or predictive biomarkers continues to remain a pressing clinical challenge. Identification of tumor specific biomarkers can lend towards an early diagnosis, expedited initiation of therapy, monitoring of therapeutic response, and early detection of recurrence of disease. We carried-out a complex analysis of cell lines and cell line derived small extracellular vesicles (sEVs) using label-free-based Quantitative Proteomic Profiling with an intent to determine shared and distinct features of these tumor cells and their respective sEVs. We analyzed EWS cells with different *EWS-ETS* fusions (*EWS-FLI1* type I, II, and III and *EWS-ERG*) and their corresponding sEVs. Non-EWS controls included osteosarcoma, rhabdomyosarcoma, and benign cells, *i.e.,* osteoid osteoma and mesenchymal stem cells. Proteomic profiling identified new shared markers between cells and their corresponding cell-derived sEVs and markers which were exclusively enriched in EWS-derived sEVs. These exo-biomarkers identified were validated by *in silico* approaches of publicly available protein databases and by capillary electrophoresis based western analysis (Wes). Here, we identified a protein biomarker named UGT3A2 and found its expression highly specific to EWS cells and their sEVs compared to control samples. Clinical validation of UGT3A2 expression in patient tumor tissues and plasma derived sEV samples demonstrated its specificity to EWS, indicating its potential as a EWS biomarker.

## 1 Introduction

In this study we applied a global approach and carried-out a systems level analysis of proteome profiles on Ewing Sarcoma (EWS) derived cell lines. After osteosarcoma, this malignancy continues to remain the second most prevalent pediatric bone malignancy (Ferguson and Turner, 2018). EWS is more common in children and teenagers but can occur in any age demographic (Balamuth and Womer, 2010). EWS is pathologically characterized by chromosomal translocation between EWS gene (chromosome 22) and members of the ETS family of transcription factors (such as FLI1, ERG, ETV1, E1AF, *etc.*) resulting in fusion oncoprotein/transcription factor (May et al., 1993). Most common fusion reported in approximately 85% of the cases is EWS-FLI1. It is a highly aggressive pediatric osseous and soft tissue malignancy thought to originate from primordial bone marrow-derived mesenchymal stem cells and consists of small round blue cells with minimal stroma and differentiation (Choi et al., 2014; Sole et al., 2021). EWS can metastasize to other bones, bone marrow, and lungs. Overt metastatic disease is prognostic; however, the preponderance of EWS patients (even with localized disease) harbor micro metastatic disease. EWS is therapeutically a very challenging form of osseous malignancy due to its pattern of metastasis, recurrence rates and lack of known druggable therapeutic targets (Uren and Toretsky, 2005; Zöllner et al., 2021). Most recurrences occur within 2 years from initial diagnosis, with a median relapse free interval of 17 months (Rodriguez-Galindo et al., 2003; Esiashvili et al., 2008). EWS has a poor survival rate in the face of metastatic disease, with no more than 10% survival of the 35% who develop recurrence. Despite the majority having of patients presenting with localized disease, approximately 30% will succumb to relapse and die despite salvage therapies. Therefore, the discovery of novel EWS biomarkers for diagnosis and monitoring disease progression and recurrence are imperative in the management of these patients. Discovery of disease specific liquid biopsy-based biomarkers could also serve as guidance in the development of novel targeted therapeutic strategies. Most currently researched biomarkers are prognostic in nature and rely upon biopsy/resection of tumor tissue; however, the mechanistic underlying origin and development of these tumors are relatively unknown.

Small extracellular vesicles (sEVs) or exosomes are a new target for liquid biopsy, which is a non-invasive method of identifying disease specific biomarkers in biological fluids such as blood, saliva, urine, cerebrospinal fluid, *etc.* (Killingsworth et al., 2021). sEVs are small lipid bilayer membrane containing vesicles with a size range of 40–160 nm in diameter (Kalluri and LeBleu, 2020) that are actively secreted by most cell types in the body including cancer cells and stably circulate in the body. sEVs are formed in the multivesicular bodies (MVB) and are released from the cells into the extracellular space following the fusion of MVBs with the plasma membrane (Mathivanan et al., 2010; Raposo and Stoorvogel, 2013). sEVs are heterogenous consisting of nucleic acids, lipids, and proteins that reflect the cargo of the cell of origin (Yanez-Mo et al., 2015). For the proteomic profiling studies presented in this manuscript, we use the term sEVs to describe vesicles recovered in the 100,000 x g centrifugation step, which are primarily 50–200 nm in size.

Specifically, EWS derived sEVs have been shown to mediate cross-talk with the surrounding tumor microenvironment and promote immunosuppressive phenotypes (Gassmann et al., 2021; Pachva et al., 2021). We have recently reported that circulating sEVs can be used to accurately diagnose EWS patients using a panel of exo-miRNAs (Crow et al., 2022). sEVs have great potential to be explored as biomarkers to detect early-stage cancers and also monitor tumor progression and predict outcomes (Xu et al., 2018; Möller and Lobb, 2020). In our previous publication, we identified several exo-protein biomarkers and demonstrated the ability to enrich EWS derived sEVs utilizing two proteins—CD99 and NGFR (Samuel et al., 2020). Through this current study we expanded our previous dataset to include proteomics data from additional EWS and non-EWS cells and their respective sEVs with the aim of identifying proteins that are enriched in sEVs that can be further developed as a liquid-based clinical test to help diagnose EWS and monitor disease burden.

We employed the latest proteomics technology and a comprehensive bioinformatic analysis to systematically characterize and compare the proteome of EWS cell lines and cell line derived sEVs and to non-EWS tumor cells such as osteosarcoma, rhabdomyosarcoma and benign cells including bone-marrow derived mesenchymal stem cells and benign osteoid osteoma cells. In an era of advanced “omics” and big data analysis, proteomics can serve as a valuable tool for investigating diversity of proteomes and gain novel insight into disease.

## 2 Material and methods

### 2.1 Experimental data set

We derived an extensive proteomic data set consisting of profiles for EWS cell lines and their corresponding sEVs. We selected a panel of confirmed cell lines which are representative of the most common *EWS-ETS* fusion types; namely, *EWS-FLI1* type I (CHLA-32, TC-32, TC-71), *EWS-FLI1* type II (RD-ES and SKES-1), *EWS-FLI1* type III (CHLA-258) fusions, as well as an *EWS-ERG* fusion (COG-E-352) (Samuel et al., 2020). For controls, rhabdomyosarcoma cell lines, CRL-2061 and TE-671, osteosarcoma cell lines, U2OS and MG-63, Hs919. T, a benign osteoid osteoma, and MSC cell line were utilized (Supplementary Table S1). MSCs, Hs919. T, SK-ES-1, RD-ES, U2-OS, MG-63, CRL-2016 and TE-671 were purchased from the American Type Culture Collection (ATCC). In addition, TC-71, TC-32, CHLA-32, CHLA-9, COG-E-352, and CHLA-258 cell lines were obtained from the Children’s Oncology Group (COG). Two biological replicates of each sEV preparations were obtained from their respective cell line derived conditioned media. Each biological replicate was analyzed as technical replicates to further establish a reliable sEV proteome (*i.e.,* exo-proteome) profile.

### 2.2 SEV isolation and cell lysate preparation

For sEV isolation, EWS cell lines were grown and cultured in their respective exosome-free FBS medium until cellular sub-confluency of ∼70% was reached. TC-71, TC-32, CHLA-32, CHLA-9, CHLA-258 and COG-E-352 were cultured in Iscove’s Modified Dulbecco’s Medium (Gibco #12440–053) supplemented with 20% FBS, 2 mM L-glutamine and 1X ITS (Gibco #41400–045). SKES-1 was cultured in McCoy’s 5A medium (ATCC #30–2007) with 10% FBS, CRL-2016 and RD-ES was cultured in RPMI-1640 (HyClone #SH30027.01) medium with 10% and 15% FBS respectively. U2OS, MG-63, TE-671 and Hs919. T were cultured in Dulbecco’s Modified Eagle’s Medium (ATCC #30–2002) with 10% FBS. Bone marrow derived MSC cells were cultured in MSC basal media (ATCC# PCS-500–030).

Conditioned media from cell culture were collected and immediately centrifuged at 2,500 rpm for 5 min to eliminate cellular debris. A total of 150 mL of conditioned medium was collected and ultra-centrifuged at 4 °C for 45 min at 8,700 rpm (10,000 × g). The supernatant was then collected and ultracentrifuged again at 4°C for 75 min at 28,800 rpm (110,000 × g). sEV pellets were washed with PBS and spun again at 4°C for 60 min at 35,800 rpm (xg) in Beckman Coulter Quik-Seal Centrifuge Tubes (Cat#). Finally, each sEV pellet was resuspended in 50 μL of PBS based on pellet size and then stored at −80°C. On an average 50–150 µg of protein was obtained from sEVs isolated from 150 mL of conditioned media (approximately 5.33 × 10^10^ particles). For isolation of cellular proteins, all the cell lines mentioned above were cultured in the similar media with regular FBS. RIPA buffer was used to isolate total proteins from the cell lines for Mass-spectrometry analysis.

### 2.3 Cell line and label-free LC-MS sEV protein quantification

EWS cell line and associated sEVs were reduced with 20 mM DTT at 60°C for 30 min and alkylated with 40 mM iodoacetamide at room temperature for 30 min. The samples were trypsinized using an S-Trap Micro Spin Column (Protifi, Farmingdale, NY). The lyophilized S-Trap eluates were redissolved into 2% acetonitrile/0.1% formic acid prior to LC-MS analysis. Tryptic peptides were separated using an EASY n-LC (ThermoFisher, Waltham, MA) UHPLC system with Acclaim PepMap 100 75 μm × 2 cm, nanoViper (C18, 3 μm, 100 Å) trap and RSLC 50 μm × 15 cm, nanoViper (C18, 2 μm, 100 Å) separating columns (ThermoFisher). The columns were heated at 50 °C. Following injection of the sample onto the column, separation was accomplished with a 50 min linear gradient from 2% acetonitrile to 37% acetonitrile in 0.1% formic acid. The eluate was introduced into the LTQ-Velos-Orbitrap ELITE + ETD mass spectrometer using a Nanospray Flex source (ThermoFisher). An Orbitrap Elite—ETD mass spectrometer (ThermoFisher) was used to collect data from the LC eluate. An *N*th Order Double Play method was created in Xcalibur v2.2. Scan event one obtained an FTMS MS1 scan (normal mass range; 120,000 resolution, full scan type, positive polarity, profile data type) for the range 300–2000 m/z. Scan event two obtained ITMS MS2 scans (normal mass range, rapid scan rate, centroid data type) on up to 20 peaks that had a minimum signal threshold of 5,000 counts from scan event one. The lock mass option was enabled (0% lock mass abundance) using the 371.101,236 m/z polysiloxane peak as an internal calibrant. Each sample was injected twice yielding essentially a technical replicate, to aid with observation and ID of low abundant proteins.

The EWS cell line and derived sEV mass spectrometry proteomics data sets were evaluated by Proteome Discoverer v2.4.0.305 (ThermoFisher) for imputation, match-between-runs, and normalization steps. Search parameters included: variable methionine oxidation; variable Acetyl, Met-loss, and Met-loss + Acetyl at the protein N-terminus; fixed cysteine carbamidomethylation; up to 2 missed tryptic cleavages; 10 ppm precursor error for MS1 Orbitrap FTMS data and 0.6 Da error for CID-based MS2 LTQ data. To estimate the false discovery rate, a decoy database was generated from this database using the Percolator node in Proteome Discoverer. For samples with peptides not confidently identified *via* Proteome Discoverer mass tolerance windows and chromatographic alignments were utilized to determine peptide presence. With presence of an m/z signal, area of the ion was extracted and utilized to populate missing values. For runs without m/z values within the mass accuracy window nor chromatographic retention time then no value was recorded. MS1 area values for peptides with high confident MS2 data were represented as high signal levels (green values). MS1 area values for peptide features that match within mass accuracy tolerance and retention time tolerance to high confidence data in a separate LC-MS run were represented as peak found but not sufficient for MS2 validation (yellow values). For non-detectable proteins without sufficient information to assign high confidence spectra (green value) nor with mass accuracy or retention time tolerances (yellow data), no signal or red value was assigned.

### 2.4 Bioinformatic analysis

Mass spectrometry proteomics data was analyzed directed by Proteome Discoverer v2.4.0.305, discovering a total of 2,038 cell line proteins and 1,294 cell line derived sEV proteins (exosomal proteins or exo-proteins).

### 2.5 Statistical analysis

Qprot v1.3.5 (Choi et al., 2015) was used to calculate Z-statistics, log2 (foldchange) and FDR (false discovery rate) values. Qprot analysis was performed with a burn in value of 2000 and 10,000 iterations. To identify differentially expressed proteins between the two groups (Ewing Sarcoma and Non-Ewing), Z-statistic values > 1.5 and FDR <0.05 or Z-statistic values < -1.5 and FDR <0.05 were selected as filter values.

### 2.6 Capillary Western blot (Wes) analysis

Capillary Western analyses were performed using the ProteinSimple Wes System. For all the analyses 0.4 μg/μL of protein sample was used. EWS cell lines, controls, and cell line derived sEV samples were diluted with 0.1× Sample Buffer. Then 4 parts of diluted sample were combined with 1 part 5x Fluorescent Master Mix (containing 5x sample buffer, 5x fluorescent standard, and 200 mM DTT) and heated at 95 °C for 5 min. The Fluorescent Master Mix contains three fluorescent proteins that act as a ‘ruler’ to normalize the distance for each capillary because the molecular weight ladder is only on the first capillary and each capillary is independent. After this denaturation step, the prepared samples, blocking reagent, 1:50 diluted primary antibodies; UGT3A2 (Fisher scientific #50–173-3388), AMER2 (Invitrogen#PA570111), GPR64 (Invitrogen# PA565594), Flotillin-1 (Cell signaling, #18634) and β-actin (Cell signaling #12262), HRP-conjugated secondary antibodies (Anti-goat and anti-mouse) and chemiluminescent substrate were dispensed into designated wells in an assay plate. A biotinylated ladder provided molecular weight standards for each assay. After plate loading, the separation electrophoresis and immunodetection steps take place in the fully automated capillary system.

### 2.7 Nano-particle tracking analysis

Size distribution and number of EVs isolated from conditioned media and patient plasma samples were analyzed with NanoSight (Malvern Instruments Ltd., Malvern, UK) and the data acquired was analyzed using NTA3.3 software suite provided with Nanosight.

### 2.8 Plasma sEV isolation and ELISA

Plasma samples (500 µL each) were diluted to 1 mL with PBS and sEVs were isolated using size exclusion columns (Exo-spin, Cell guidance systems, Cat# EX04-20) per the protocol provided by the manufacturer. The sEVs were eluted in 3 mL PBS and further concentrated to 1 mL using Amicon Ultra-4 centrifugal filters (Millipore, Ultracel-10K, Cat# UFC801024) per directions from the manufacturer. Obtained sEV samples were stored at −80C for further use. On an average, sEVs isolated from 500 µL of plasma resulted 1.6 μg/μL of protein (from approximately 4.05 × 10^11^ particles) which was used for further analysis. UGT3A2 ELISA was performed with plasma-derived sEVs using commercially available kit (MyBiosource.com, human UGT3A2, #MBS9336154) per the directions provided by supplier. For this, 100 µL of plasma derived sEVs were lysed using 50X cell extraction enhancer (Abcam #6300004) and 5X extraction buffer (Abcam #6300003). Absorbance was determined using Tecan plate reader. Absorbance was normalized to control wells and UGT3A2 concentration in sEV samples were determined by interpolating their absorbances from standard curve using Prism 9 (ver 9.0.0) software (GraphPad, San Diego CA). Statistical analysis was performed using Mann-Whitney test in GraphPad Prism 8 software where *p* < 0.05 was considered statistically significant.

### 2.9 Immunohistochemistry (IHC)

Immunohistochemistry staining of 5-μm-thick formalin-fixed paraffin-embedded EWS and Rhabdomyosarcoma tissue sections was performed using anti‐UGT3A2 antibody (Proteintech, #21366-1-AP). Briefly, after deparaffinization and rehydration of the slides, antigen retrieval was performed using Citrate Buffer-pH 6.0 (Sigma Aldrich, Cat No #C9999). BLOXALL Endogenous Blocking Solution (Vector laboratories, Cat No# SP-6000–100) was used to block non-specific binding. All the slides were then incubated overnight with primary antibody at 4°C. Following incubations, sections were washed with PBS and incubated for an hour with ImmPRESS HRP Horse anti-rabbit secondary antibody (Vector laboratories, Cat# MP-7801–15). Finally, immunoreactivity was detected using DAB Substrate Kit (Vector laboratories, Cat No# SK-4100) per manufacturer instructions. Stained sections were reviewed by a pathologist using high resolution light microscopy. The H-scores were calculated based on staining intensities (ranging from 0: no staining, 1+: weak, 2+: moderate and 3+: strong staining) using the formula: H-Score = (0 x percentage of cells with absent cytoplasmic staining) + (1 x percentage of “1+" cells) + (2 x percentage of “2+" cells) + (3 x percentage of “3+ cells). Statistical analysis was performed using t-test where *p* < 0.05 was considered statistically significant, and data was plotted in GraphPad Prism 8 software.

## 3 Results

### 3.1 Identification of Enriched sEV protein markers for Ewing Sarcoma

**FIGURE 1:**
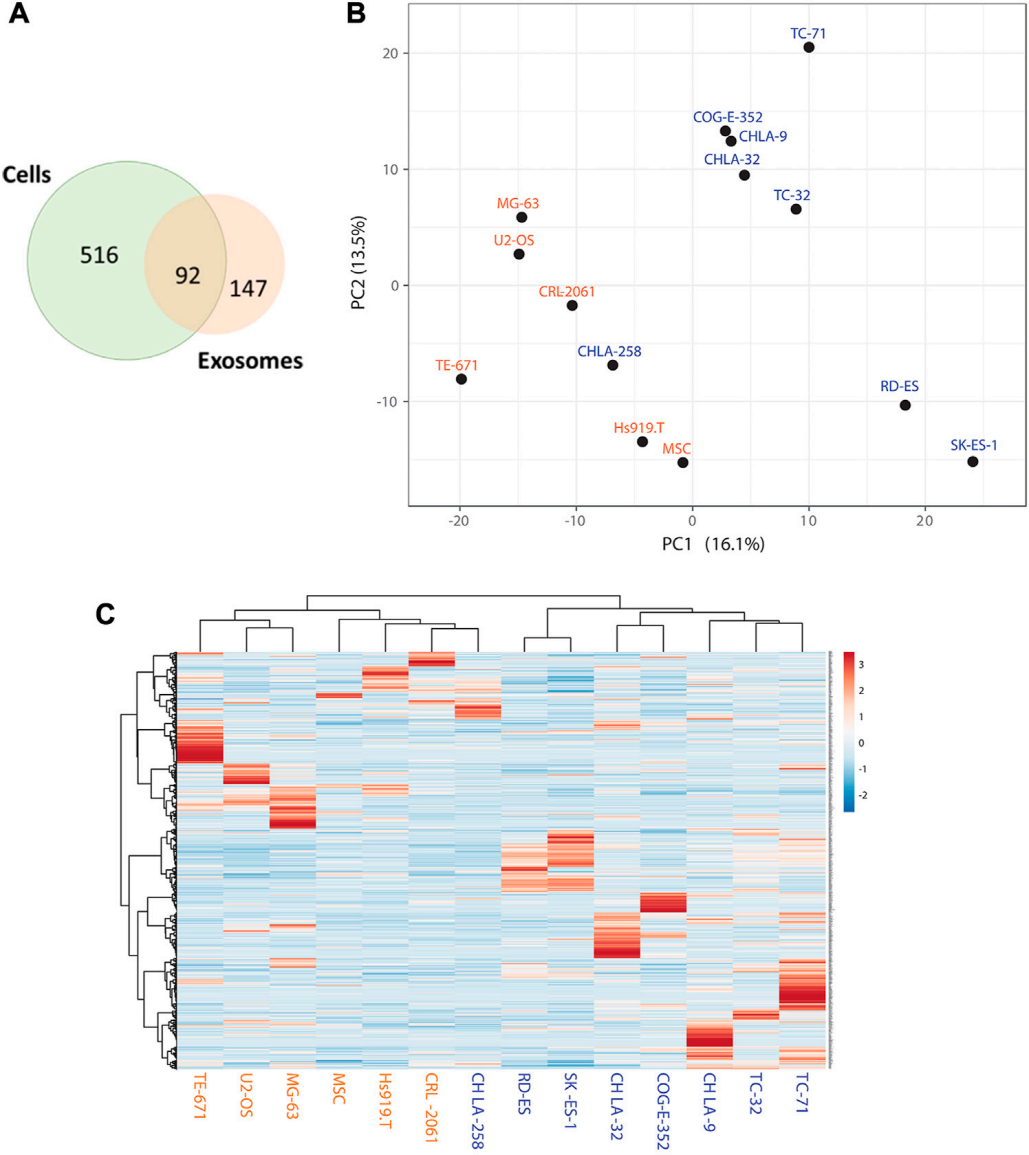
Identification of Enriched sEV Proteins for EWS by Mass-spectrometry **(A)** Venn diagram representing total number of proteins identified in EWS cells and sEVs and overlap of shared proteins between these two groups. **(B)** Principal Component Analysis (PCA) plot illustrating relatedness among the samples based on 1,294 protein expressions. The first two principal components are shown, illustrating the degree of similarity between the samples. *X* and *Y*-axis show principal component 1 and principal component 2 with 16.1% and 13.5% of the total variance, respectively. **(C)** Hierarchical clustering on all 14 samples and 1,294 proteins. Rows are centered; unit variance scaling is applied to rows. Both rows and columns are clustered Pearson correlation as a distance and average as a method. Blue and orange color labels indicate EWS and non-EWS cells respectively.

**FIGURE 2:**
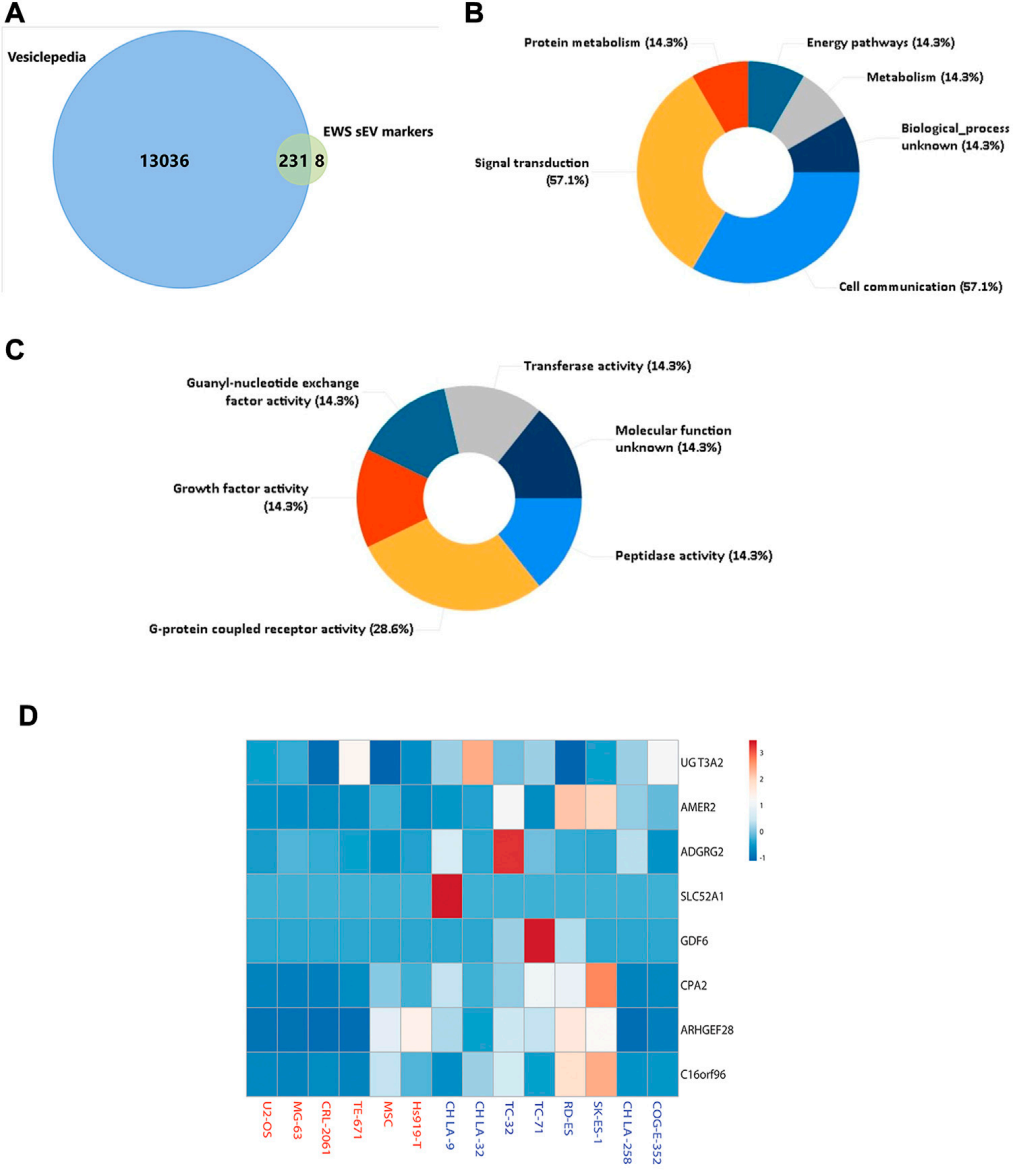
Characterization of EWS Specific sEV (exo-) Proteins **(A)** Venn diagram representing proteins in Vesiclepedia and sEVs, eight unique proteins were identified in EWS sEVs. **(B)** Top-ranked over-represented biological processes and **(C)** predicted molecular functions for the eight EWS exo-protein markers. **(D)**. Heat map representation for expression of eight EWS exo-protein biomarkers using quantitative values. Rows are centered; unit variance scaling is applied to rows.

We performed mass-spectrometry analysis of cell lines and their associated sEVs from different types of EWS cells bearing *EWS-FLI1* (Type I-III) and *EWS-ERG* fusion and compared them to non-EWS cells as specificity controls (Supplementary Table S1). We identified exclusively enriched 608 proteins in EWS cell lines and 239 proteins in EWS sEVs (exo-proteins) compared to non-EWS cells and their sEVs. 92 proteins were found to be shared between EWS cells and their corresponding sEVs (Figure 1A). Principal component analysis (Figure 1B) and hierarchical clustering (Figure 1C) of exo-proteins identified distinct biomarkers that could distinguish EWS from non-EWS. In comparison, hierarchical clustering of the proteins identified from EWS, and non-EWS cell lines does not separate the cell of origin (Supplementary Figure S1). Of the 239 EWS exo-proteins, eight were uniquely enriched (Supplementary Table S2) and not previously reported within Exocarta (Simpson et al., 2012) (exocarta.org) and Vesiclepedia (Kalra et al., 2012); a molecular database of published extracellular vesicle studies (Figure 2A). In the cell, most of these proteins are associated with signal transduction and cell-cell communication (Figure 2B). We determined the predicted molecular functions of these enriched exo-proteins by using FunRich (Functional Enrichment analysis tool) (Pathan et al., 2015) (Figure 2C); with predicted involvement in G-protein coupled activity (GPR64/ADGRG2, SLC52A1), growth factor activity (AMER2, GDF6), transferase activity (UGT3A2), guanyl-nucleotide exchange factor activity (ARHGEF28) and peptidase activity (CPA2). A heatmap plot of the expression of these eight EWS- associated exo-proteins indicate higher abundance in EWS-sEVs as compared to non -EWS sEVs (Figure 2D).

### 3.2 Identification of shared proteins between EWS cells and sEVs

**FIGURE 3:**
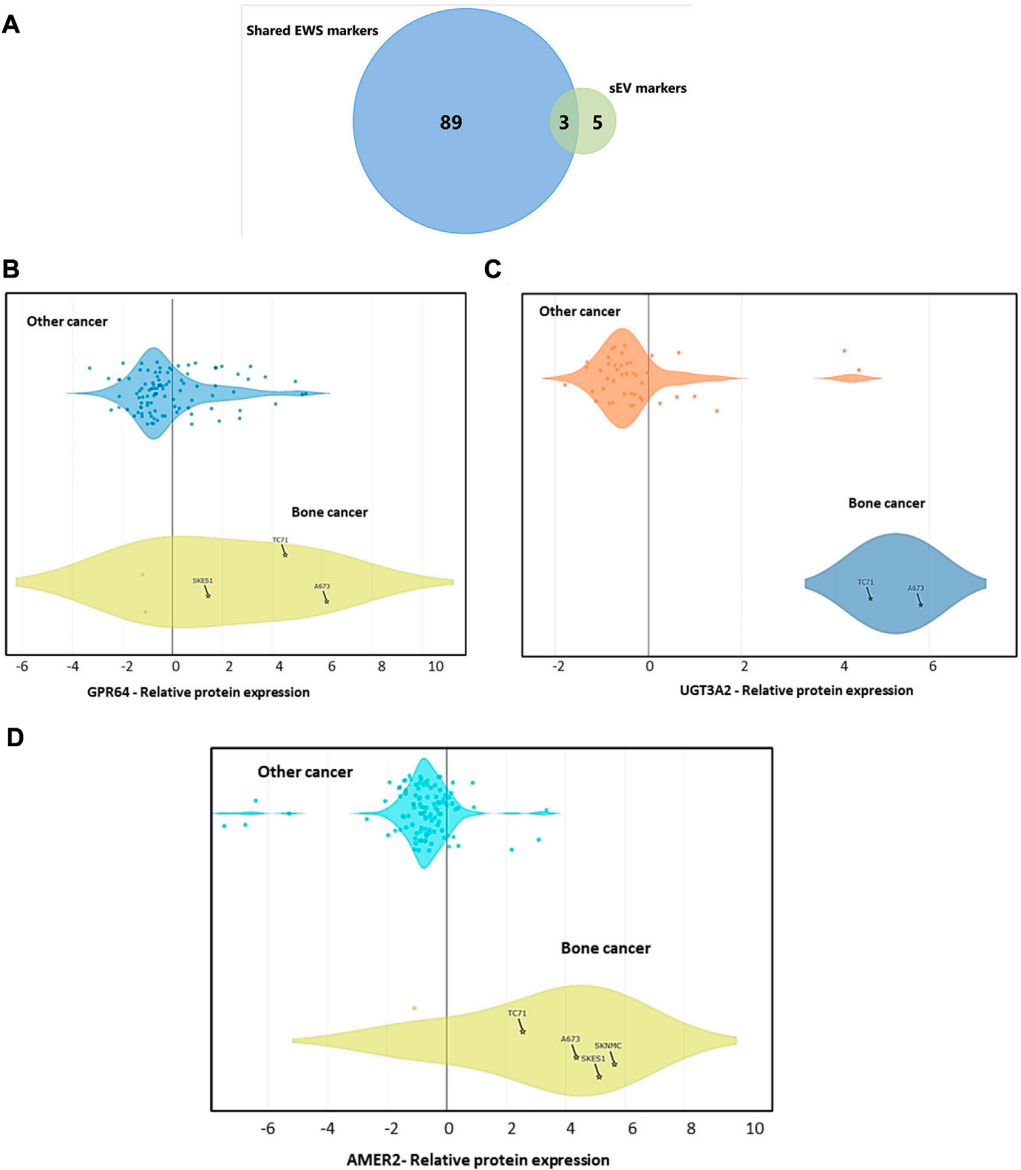
Protein Expression Data Across Different Cancer Cell Lines from DepMap Portal **(A)** Shared markers between cells and sEVs. Data explorer tool and proteomics database of DepMap portal has been used to generate violin plots representing the protein expression of **(B)** GPR64, **(C)** UGT3A2, and **(D)** AMER2 compared to other cancer types.

Utilizing Exocarta (exocarta.org) and Vesiclepedia we further screened the 92 shared proteins between EWS cells and their corresponding sEVs and found that there were only three exo-proteins, UDP glycosyltransferase 3A2 (UGT3A2), APC membrane recruitment 2 (AMER2), and G-protein coupled receptor 64 (GPR64/ADGRG2) which are commonly enriched in both EWS cells and cell-derived sEVs (Figure 3A). We further investigated these proteins using the Dependency Map (DepMap) portal (https://depmap.org/portal/) to assess the expression of top-8 EWS exo-proteins. We observed that relative to other cancer types, protein expression of GPR64 (Figure 3B), UGT3A2 (Figure 3C) and AMER2 (Figure 3D) is typically enhanced in EWS cancer cells (Supplementary Figure S2). For the other prioritized proteins, no difference in expression was seen compared to other cancer types (Supplementary Figure S3), hence these markers were not further validated in this study. Similarly, we were also able to evaluate mRNA expression of the genes encoding these three protein biomarkers *via* BioGPS (biogps.org) and found the expression of GPR64 and AMER2 in EWS patient tumor samples to be consistent regardless of stage of disease, whereas UGT3A2 expression was not available in this dataset (Supplementary Figure S4).

### 3.3 Validation of protein expression by Wes Analysis

**FIGURE 4:**
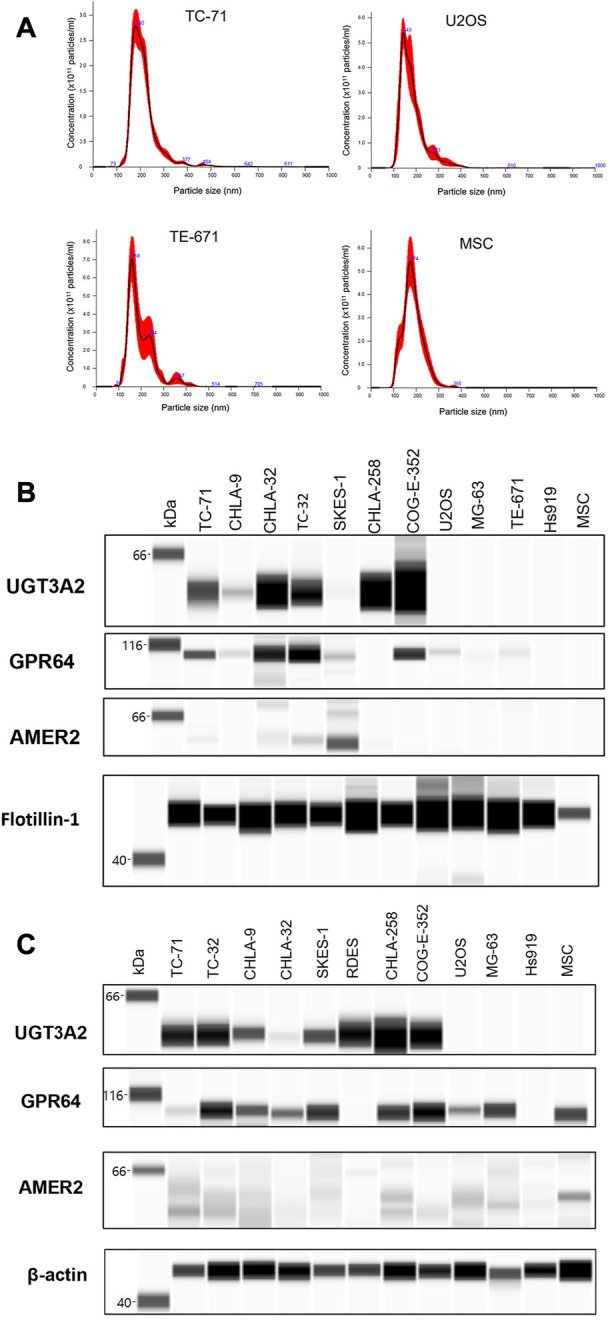
Validation of EWS cell and sEV Protein Expression by Wes Analysis **(A)** Representative NTA analysis plots for cell-derived sEVs, indicating the particle size range between 100–200 nm. Wes analysis for UGT3A2, GPR64 and AMER2 protein expression in **(B)** cell-derived sEVs and **(C)** cell lines.

We next validated our mass spectrometry data of our top shared protein biomarkers by capillary electrophoresis based simple western (Wes) analysis. sEV samples isolated from conditioned media of cells were further characterized by nano-particle tracking analysis (NTA). sEVs isolated were between the size ranges 100–200 nm and the representative NTA graphs are presented to show particle size distribution in isolated sEV samples (Figure 4A; Supplementary Figure S5). Cell-line derived sEVs isolated by differential ultracentrifugation (Figure 4B) and their parental cell lines (Figure 4C) from different EWS and non-EWS samples were used to validate protein expression. UGT3A2 expression was observed exclusively in all EWS cell lines and their respective sEVs lysates as compared to non-EWS control cells and sEVs with no UGT3A2 expression. GPR64 expression was not exclusive to EWS cells and was observed in control cells and sEVs as well, whereas AMER2 protein expression was very low/undetecable in EWS cells and only the sEVs from SKES-1 EWS cells showed detectable AMER2 levels. Based on the Wes validation and quantification studies (Supplementary Figure S6), we focused on UGT3A2 that was highly specific to EWS cells and their sEVs compared to other markers and used it for further clinical validation studies.

### 3.4 Higher UGT3A2 expression is detected in EWS patient samples

**FIGURE 5:**
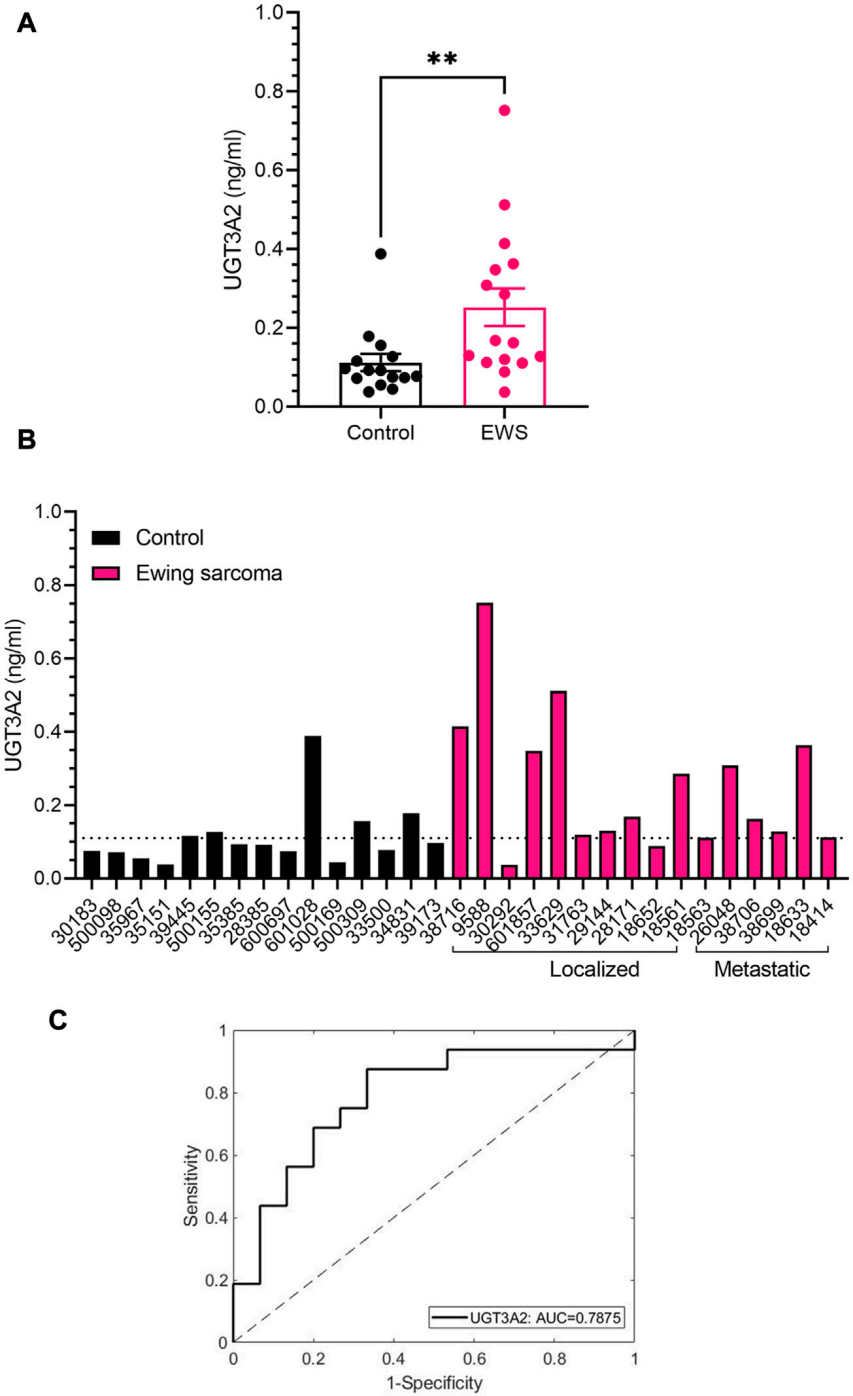
Clinical Validation of UGT3A2 Expression in Patient Plasma and Tumor Sections **(A)** UGT3A2 expression in control and EWS patient plasma-derived sEV samples as determined by ELISA. **(B)** Individual expression of UGT3A2 in healthy controls and EWS patient samples (localized and metastatic). Significant difference in expression was observed between healthy controls and EWS patients (**p = 0.0055 assessed by Mann-Whitney test). **(C)** ROC curve for UGT3A2 ELISA. The black line shows the mean area under the curve (AUC) plot, with the AUC value of 0.7875.

**FIGURE 6:**
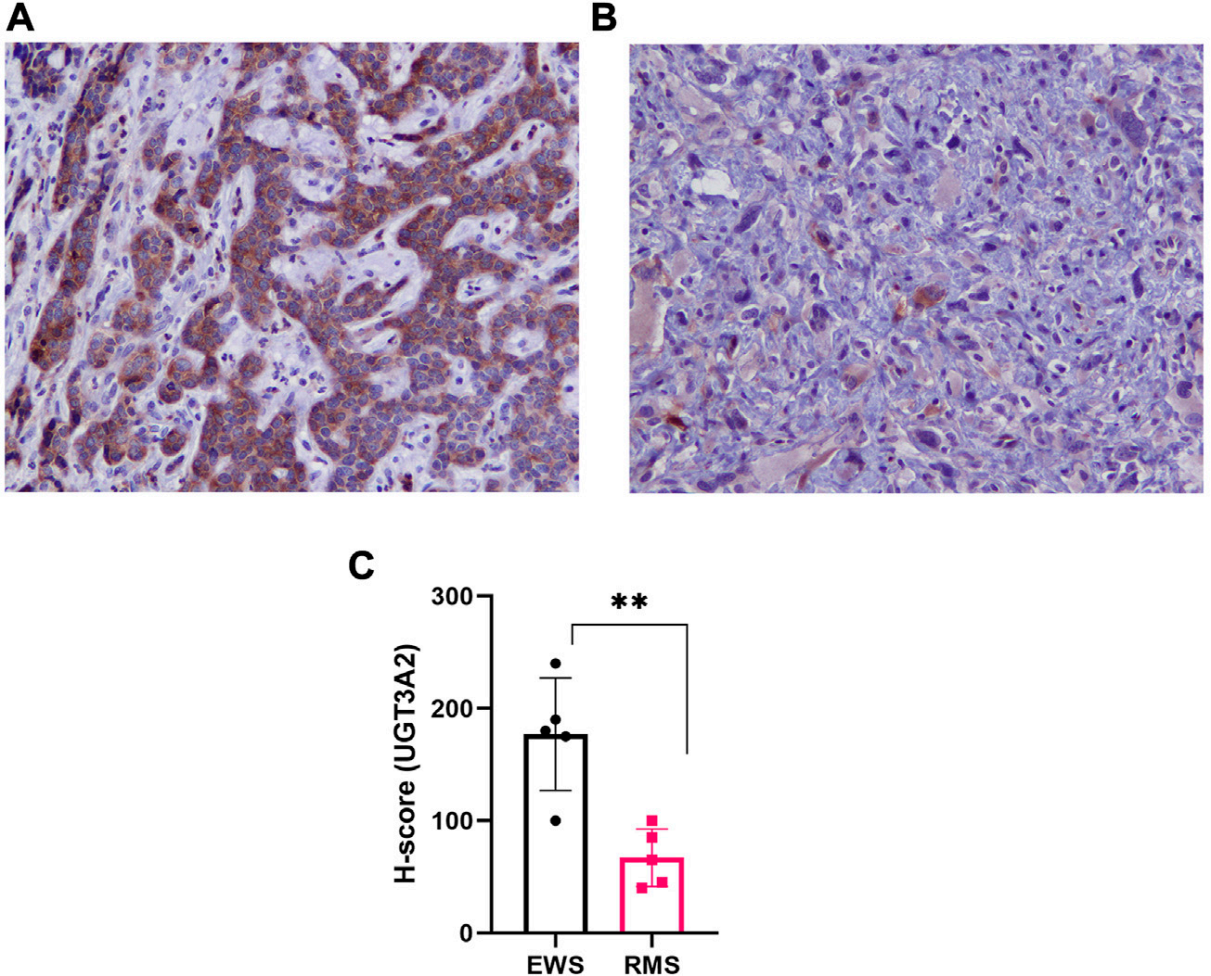
Immunohistochemical Staining for UGT3A2 Expression in Patient Tumor Sections. Representative IHC (20X images) of representative patient tumor tissue sections indicating strong UGT3A2 staining in **(A)** EWS compared to **(B)** Rhabdomyosarcoma samples. **(C)** H-score was calculated for EWS (*n* = 5), and RMS (*n* = 5) patient tissue sections based on UGT3A2 expression and is represented as bar graph. EWS samples demonstrated higher expression of UGT3A2 as compared to RMS tumor sections (**p = 0.0024 assessed by t-test).

Based on our *in silico* (Figure 3C) analysis and Wes validation (Figure 4B), we evaluated UGT3A2 protein expression in plasma-derived sEVs from EWS patients (both localized and metastatic disease) and age-matched healthy controls (Supplementary Table S3). We observed that UGT3A2 expression is significantly higher in the EWS patient samples (Figures 5A, B) resulting in an AUC = 0.7875 with a 95% confidence interval (CI) of 0.6202–0.9548 (*p*-value = 0.0004). The corresponding Receiver operating characteristic (ROC) plot is given in (Figure 5C). No difference in UGT3A2 expression was observed when we separated EWS patients based on stage of disease (localized vs. metastatic) (Supplementary Figure S7). When compared to our previously reported EWS sEV-associated biomarkers (Samuel et al., 2020) Ezrin, CD99, NGFR and ENO2, we explored the complementary potential of UGT3A2 in combination with the other markers (Supplementary Table S4; Supplementary Table S9). By using the linear term of the associated logistic regression models to create combination scores of UGT3A2 with each of the other markers, we obtain the ROC results that are reported in Supplementary Table S5. We see that there is evidence for further improving the accuracy up to an AUC of 0.9792 when combining UGT3A2 with Ezrin to obtain high specificity and sensitivity. Overall, these results provide evidence of the complementary nature of the discussed markers in attaining high discriminatory ability and indicate the potential of UGT3A2 to be leveraged as a liquid biopsy-based biomarker for EWS. Further, immunohistochemistry (IHC) for UGT3A2 expression in representative EWS (Figure 6A) and Rhabdomyosarcoma (Figure 6B) patient tissue sections reveals stronger staining in EWS samples. We analyzed tumor sections from 5 patients for each tumor type and calculated H-scores that indicated stronger expression of UGT3A2 in EWS samples compared to RMS (Figure 6C). Together, these data suggest that UGT3A2 can be used as a potential diagnostic biomarker for EWS**.**

## 4 Discussion

Ewing sarcoma is a rare and aggressive form of pediatric cancer affecting children and young adults. The current multi-modal treatment options have improved the survival rate of patients with non-metastatic disease, whereas for patients with refractory, recurrent and metastatic disease the survival rates remain dismal. It is essential to understand genomic and proteomic alterations seen in patients before and after treatment to predict response to therapy as well as identify potential markers that could predict tumor recurrence or metastasis. Furthermore, proper pathologic diagnosis of EWS often include invasive methods including tumor biopsy. Identification of circulating tumor biomarkers on sEVs, cell free (cf)DNA/circulating tumor (ct)DNA based molecular techniques can help identify tumor specific markers in a non-invasive and rapid manner. The main goal of this study is to identify protein markers that are specifically enriched in EWS sEVs that can be further validated and developed as EWS disease liquid biopsy markers.

We utilized mass spectrometric technology and robust computational methods to characterize the proteome of EWS cell lines and their respective sEVs. We used a stringent approach and included several different *EWS-ETS* fusion bearing cell lines and their corresponding sEVs for the discovery screen and compared them to non-Ewing’s and benign control cell lines. Patient clinical samples were used for validation studies. We further screened the identified proteins *via* publicly available databases, including Vesiclepedia which helped eliminate non-specific/common sEV biomarkers. We narrowed down our list to 8 protein biomarkers specific to EWS, among which three, *i.e.,* UGT3A2, GPR64, AMER2 were enriched in both cells and sEVs. Wes protein expression validation indicated UGT3A2 as highly specific EWS cells and their sEVs with no expression in any controls used in the study. Hence, we further evaluated the potential of UGT3A2 as a biomarker in the clinical validation studies in patient tumor sections and plasma-derived sEV samples.

UGT3A2 is involved in drug metabolism (Hu et al., 2021) and was previously found to be enriched in EWS tissue samples (Annalora et al., 2018) whereas we are reporting its expression in EWS sEVs for the first time. GPR64 has known roles in other cancers (Balenga et al., 2017; Ahn et al., 2019) and has been identified as a potential target for antibody based therapy for EWS and other sarcomas (Nakamura et al., 2022). AMER2 has been previously shown to be associated with microtubule stability (Pfister et al., 2012a) and as a negative regulator of Wnt-pathway (Pfister et al., 2012b) but its role in other cancers and EWS is currently not known. We next leveraged publicly available databases such as DepMap portal (Figure 3; Supplementary Figure S2, 3) and BioGPS (Supplementary Figure S4) to explore the expression of these protein biomarkers and genes across multiple tumor cell lines and patient RNA expression data, respectively. We also compared our current mass-spectrometry data to our previously published work and identified 12 shared exo-protein biomarkers (Supplementary Figure S8) (Samuel et al., 2020). The top exo-protein biomarkers we previously reported, *i.e.,* CD99, NGFR, EZR and ENO2, plus UGT3A2 were validated in our expanded screening panel (Supplementary Figure S9) and shown to be highly informative for diagnosing EWS. Given the heterogeneity of patient population and the tumor itself, the rationale to combine new and old markers is to leverage both datasets to identify better biomarkers for detection of EWS.

The main intent of this work was to extensively profile the proteome of sEVs derived from EWS cell lines and then verify if any of these exo-proteins could be detected in circulating EVs population. We have used 500 µL of patient plasma samples which is a feasible sample volume for sEV isolation in clinical diagnostic studies. Given that it is a very rare cancer, our expectation is the sensitivity of these exo-protein biomarkers reported in this proteomic discovery study will be further enhanced by immuno-enrichment approaches on EV microfluidic platforms (unpublished data, validation in process) that require much less clinical material (<50 µL as opposed to 500 µL in the current study).

Identification of sEV markers that can be easily detected by non-invasive liquid biopsy approaches are the need of the hour for detection of disease and monitoring tumor progression. We also have demonstrated the ability to develop microfluidic diagnostics platforms which target exo-proteins on the surface of captured sEVs (Zhang et al., 2018; Zhang et al., 2019). Future studies will explore the potential role for these exo-protein biomarkers in tracking EWS disease progression by investigating its expression in longitudinal samples collected from EWS patients pre- and post-therapy. We were able to use integration of multiple sources and use a powerful and intuitive visualization tool to extract new knowledge from the data. Such approach enables rapid discovery and evaluation of meaningful patterns hidden in multidimensional biological dataset and leads to extraction of previously unrevealed biological insights. These analyses can guide researchers in future follow-up studies for biological discovery in diagnostics and prognostics.

## 5 Data sharing

Proteomic files have been deposited in MassIVE (http://massive.ucsd.edu/) under MassIVE MSV000090623 for a study entitled “Identification of small extracellular vesicle protein biomarkers of pediatric Ewing Sarcoma”. The uploaded data for 14 cell line lysates and corresponding cell line sEV with each sample analyzed as technical duplicates. Files uploaded include (A) the primary data files (.RAW), (B) peak list files (.mzML), (C) sample key, (D) the sequence databases (02/10/2021 versions for the UniprotKB reviewed reference proteome, and (E) pdResults files for cell lysates and sEV data exported from Proteome Discoverer v2.4.0.305 (ThermoFisher) containing the SequestHT protein assignments filtered for 1% FDR.

## Data Availability

The datasets presented in this study can be found in online repositories. The names of the repository/repositories and accession number(s) can be found below: ftp://MSV000090623@massive.ucsd.edu, MSV000090623.
