# Supplementary material for: Identification of small extracellular vesicle protein biomarkers for pediatric Ewing Sarcoma
Source: Front Mol Biosci. 2023 Apr 13;10:1138594. doi: 10.3389/fmolb.2023.1138594 (PMC10140755; doi:10.3389/fmolb.2023.1138594)
Supplement: Supplementary file 1 [file DataSheet2.pdf]

SUPPLEMENTARY DATA

Supplementary Table Legends

Supplementary Table 1. List of cell lines used for mass-spectrometry. Type of cell line used and phase of therapy when the cell lines were obtained is mentioned.

Supplementary Table 2. List of 8 protein biomarkers enriched in EWS sEVs. Accession number, protein symbol, name, and cellular localization for each of the top-hits are represented

Supplementary Table 3. Patient sample information used for ELISA assays. We analyzed a total of 16 EWS patients (localized disease (n=11) and metastatic disease (n=5)) and 15 healthy controls.

Supplementary Table 4. ELISA ROC curve summary for individual markers indicating their AUC values, sensitivity and specificity data. All the biomarkers are reported to have an AUC in the range of 0.7-0.9 indicating their potential to be further explored for disease detection.

Supplementary Table 5. ELISA ROC curve summary for UGT3A2 in combination with other EWS biomarkers indicating their AUC values, sensitivity and specificity data. There is a slight improvement in AUC values when UGT3A2 was combined with ENO2. Whereas for the rest of the markers NGFR, EZRIN and CD99 AUC values remain unchanged.

Supplementary Figure Legends

Supplementary Figure 1. Hierarchical Clustering of Proteins Identified in Cell Lines. Shown is the hierarchical clustering on all 14 samples and 2,039 proteins. Rows are centered; unit variance scaling is applied to rows. Both rows and columns are clustered Pearson correlation as a distance and average as a method.

Supplementary Figure 2. DepMap Portal Protein Expression Data. Data explorer tool of DepMap portal was assessed to generate violin plots representing the protein expression of **A**) GPR64, **B**) AMER2, and **C**) UGT3A2 compared to other cancer types.

Supplementary Figure 3. DepMap Portal Protein Expression Data. Data explorer tool of DepMap portal was assessed to generate violin plots representing the RNA or protein expression of **A**) ARHGEF28 **B**) C16orf96 **C**) SLC52A1 D) CPA2 and E) GDF6 compared to other cancer types.

Supplementary Figure 4. RNA Expression of Identified sEV Protein Biomarkers in Patient Samples (BioGPS). RNA expression data of **A**) GPR64 and **C**) AMER2 from publicly available Ewing sarcoma patients (n=37) data (https://www.ebi.ac.uk/biostudies/arrayexpress/studies/E-GEOD-12102) accessed from BioGPS (biogps.org). No difference in expression of **B**) GPR64 and **D**) AMER2 was observed in patients when segregated based on stage of disease such as primary tumor (no evidence of disease), metastasis, and relapse. UGT3A2 expression was not available from this dataset.

Supplementary Figure 5. Nanotracking Particle Analysis. NTA analysis plots for cell-derived sEVs from **A**) TC-32, **B**) CHLA-32, **C)** SK-ES-1 **D**) RD-ES, **E**) CHLA-258, **F**) COG-E-352, **G**) MG-63 and H) Hs919, indicating the particle size range of sEVs between 100-200 nm. All the cell-derived sEVs were analyzed by NTA before performing additional functional studies.

Supplementary Figure 6. Wes protein expression data quantification.
Quantification data for cell-derived sEV protein expression of A) UGT3A2, B) GPR64 and C) AMER2 and cell lines protein expression of D) UGT3A2 and E) GPR64. Area of the bands was used for protein quantification and was determined by the integrated analysis tool in Wes Compass software. Lane normalization factor was calculated using the following formula: Observed signal of housekeeping protein (β-Actin or Flotillin-1) for each lane/Highest observed signal of housekeeping protein on the blot. Protein expression or normalized experimental signal for each protein/sample was calculated as Observed experimental signal/Lane normalization factor.

Supplementary Figure 7. UGT3A2 expression in patient sEV samples. ELISA was done on healthy controls and EWS patient (localized and metastatic) plasma derived sEV samples. Significant difference in UGT3A2 expression was observed between control and localized EWS patient samples (p=0.0356*), but no significant differences were observed between control vs EWS metastatic patients and localized vs metastatic patients. p-value was measured by one-way ANOVA (Tukey's multiple comparison test).

Supplementary Figure 8. Overlap of Proteins from Previous Screen. A) Venn diagram illustrating the comparison of proteins shared or new as compared to our previously published mass-spectrometry dataset. **B**) List of 12 common exo-protein biomarkers shared between the datasets.

Supplementary Figure 9. ROC curve based on ELISA for EWS Biomarkers. ROC curves for previously reported and new EWS biomarkers are represented. The colored lines represent different biomarkers and their mean area under the curve (AUC) plot, with the AUC value.
