# Supplementary material for: Identification of small extracellular vesicle protein biomarkers for pediatric Ewing Sarcoma
Source: Front Mol Biosci. 2023 Apr 13;10:1138594. doi: 10.3389/fmolb.2023.1138594 (PMC10140755; doi:10.3389/fmolb.2023.1138594)
Supplement: Supplementary file 2 [file DataSheet3.PDF]

Supplementary Table 1. List of cells lines used for Mass-spectrometry

Cell lines	Type	Phase of therapy
TC-71	Ewing Sarcoma, Type I EWS-FLI1 fusion	Post-chemotherapy
TC-32	Ewing Sarcoma, Type I EWS-FLI1 fusion	Diagnosis
CHLA-9	Ewing Sarcoma, Type I EWS-FLI1 fusion	Diagnosis
CHLA-32	Ewing Sarcoma, Type I EWS-FLI1 fusion	Diagnosis
SKES-1	Ewing Sarcoma, Type II EWS-FLI1	Unknown
RD-ES	Ewing Sarcoma, Type II EWS-FLI1	Unknown
CHLA-258	Ewing Sarcoma, Type III EWS-FLI1	Post-chemotherapy
COG-E-352	Ewing Sarcoma, EWS-ERG fusion	Post-chemotherapy
U2OS	Osteosarcoma	Unknown
MG-63	Osteosarcoma	Unknown
TE-671	Rhabdomyosarcoma	Unknown
CRL-2061	Rhabdomyosarcoma	Unknown
Hs919.T	Benign osteoid osteoma	N/A
MSC	Bone-marrow derived Mesenchymal Stem cells	N/A

Supplementary Table 2. List of identified EWS specific sEV markers

Accession number	Symbol	Protein	Cellular Localization
**Q3SY77**	**UGT3A2**	**UDP Glycosyltransferase Family 3 Member A2**	**Cytoplasm**
**Q8IZP9**	**ADGRG2/ GPR64**	**adhesion G protein-coupled receptor G2**	**Plasma Membrane**
**Q8N7J2**	**AMER2**	**APC membrane recruitment protein 2**	**Plasma Membrane**
Q9NWF4	SLC52A1	solute carrier family 52 member 1	Plasma Membrane
Q8N1W1	ARHGEF28	Rho guanine nucleotide exchange factor 28	Cytoplasm
A6NNT2	C16orf96	chromosome 16 open reading frame 96	Other
P48052	CPA2	carboxypeptidase A2	Extracellular Space
Q6KF10	GDF6	growth differentiation factor 6	Extracellular Space

Supplementary Table 3. Patient sample information

Sample	ID#	Age (Years)	Gender	Localized/ Metastatic
EWS	033629	6	F	Localized
EWS	031763	13	F	Localized
EWS	029144	1	F	Localized
EWS	028171	17	F/M	Localized
EWS	018652	17	M	Localized
EWS	018561	16	F	Localized
EWS	018563	12	F	Localized
EWS	038716	20	M	Localized
EWS	009588	19	M	Localized
EWS	030292	15	M	Localized
EWS	601857	20	M	Localized
EWS	026048	33	M	Metastatic
EWS	038706	12	F	Metastatic
EWS	038699	17	F	Metastatic
EWS	018633	16	F	Metastatic
EWS	018414	37	M	Metastatic
Healthy	030183	39	M	N/A
Healthy	500098	20	M	N/A
Healthy	035967	38	M	N/A
Healthy	035151	29	M	N/A
Healthy	039445	21	M	N/A
Healthy	500155	30	M	N/A
Healthy	035385	20	F	N/A
Healthy	028385	27	F	N/A
Healthy	600697	27	M	N/A
Healthy	601028	27	M	N/A
Healthy	500169	23	F	N/A
Healthy	500309	24	F	N/A
Healthy	033500	18	F	N/A
Healthy	034831	18	F	N/A
Healthy	039173	18	F	N/A

Supplementary Table 4. ELISA ROC curve summary for individual markers

Biomarker	AUC	SE	CI low	CI upp	p-value 1t	Sensitivity at 95% Specificity	Specificity at 95% Sensitivity
NGFR	0.9417	0.0433	0.8568	1.0000	0.0000	0.5625	0.8667
ENO2	0.8563	0.0683	0.7224	0.9901	0.0000	0.4375	0.4000
CD99	0.9792	0.0213	0.9374	1.0000	0.0000	0.7500	0.8667
EZRIN	0.9750	0.0265	0.9230	1.0000	0.0000	0.6250	0.9333
UGT3A2	0.7875	0.0854	0.6202	0.9548	0.0004	0.1875	0.0000

Supplementary Table 5. ELISA ROC curve summary for UGT3A2 in combination with other EWS biomarkers

Biomarker	AUC	SE	CI low	CI upp	p-value 1t	Sensitivity at 95% Specificity	Specificity at 95% Sensitivity
UGT & NGFR	0.9375	0.0448	0.8497	1.0000	0.0000	0.5000	0.8000
UGT & ENO2	0.9083	0.0519	0.8067	1.0000	0.0000	0.5000	0.6667
UGT & CD99	0.9792	0.0200	0.9400	1.0000	0.0000	0.8125	0.8000
UGT & EZRIN	0.9792	0.0231	0.9339	1.0000	0.0000	0.6875	0.9333
