# Supplementary material for: Identification of small extracellular vesicle protein biomarkers for pediatric Ewing Sarcoma
Source: Front Mol Biosci. 2023 Apr 13;10:1138594. doi: 10.3389/fmolb.2023.1138594 (PMC10140755; doi:10.3389/fmolb.2023.1138594)

Supplementary Figure 1. Hierarchical clustering of proteins identified in cell lines

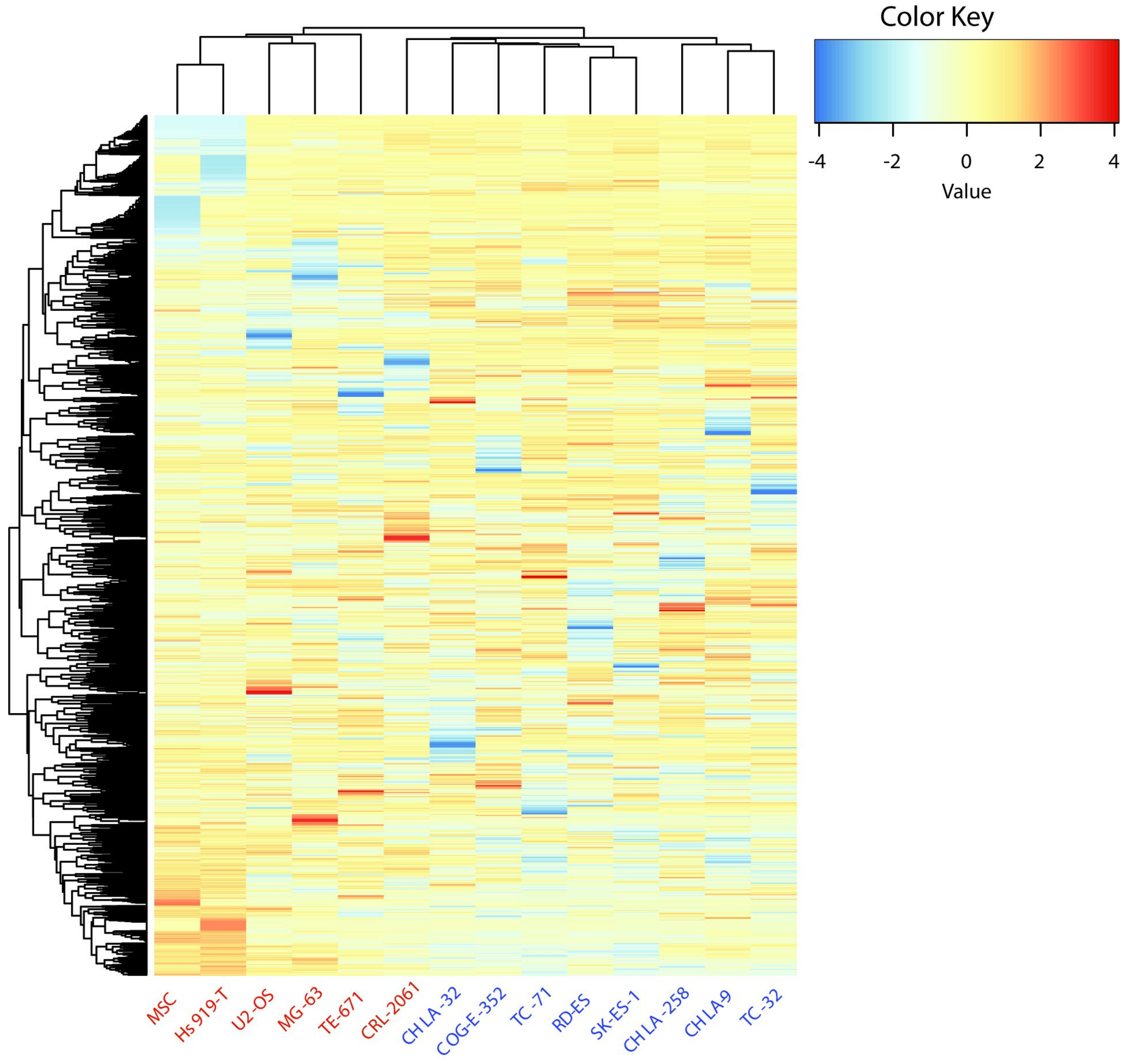

Supplementary Figure 2. DepMap portal expression data

A

GPR64

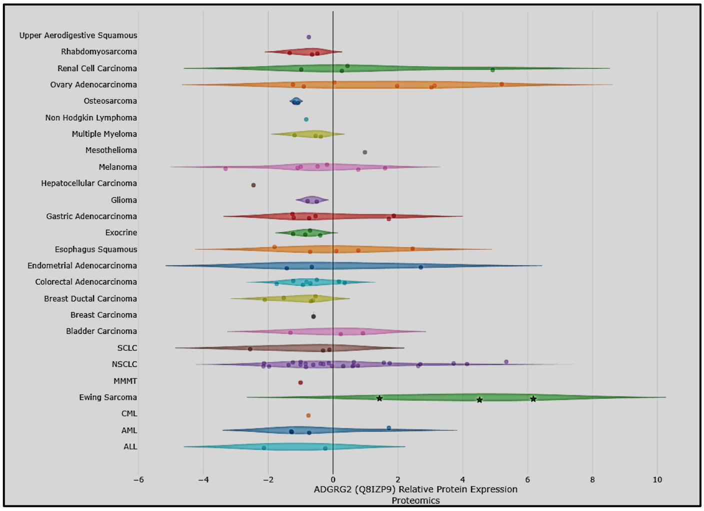

B

AMER2

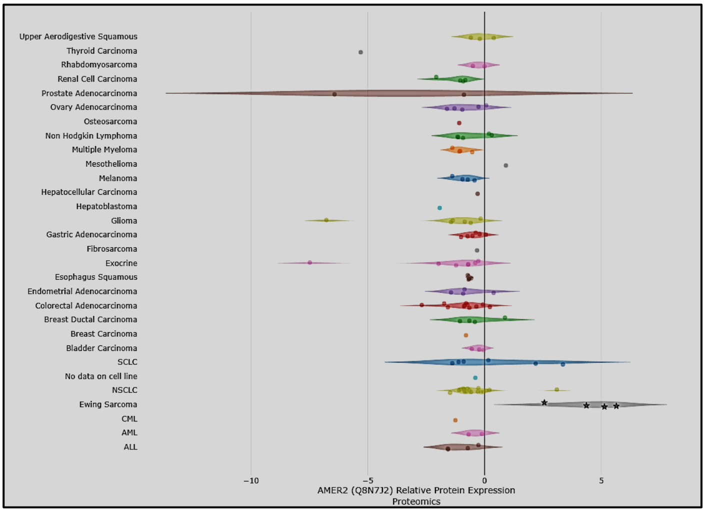

C

UGT3A2

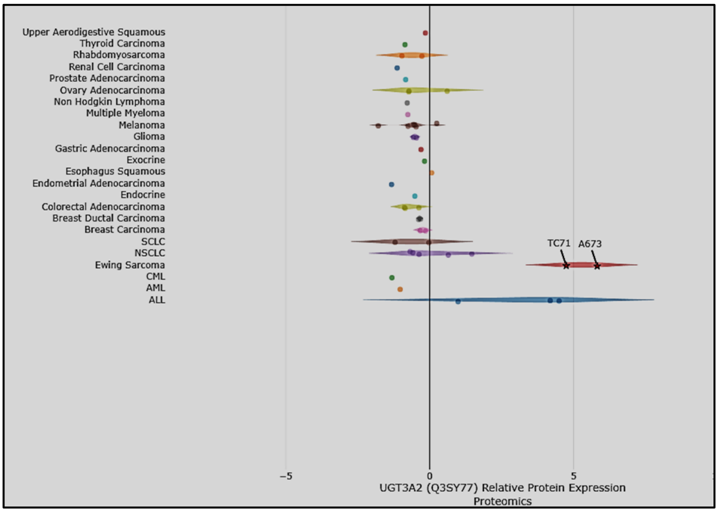

Supplementary Figure 3. DepMap portal expression data

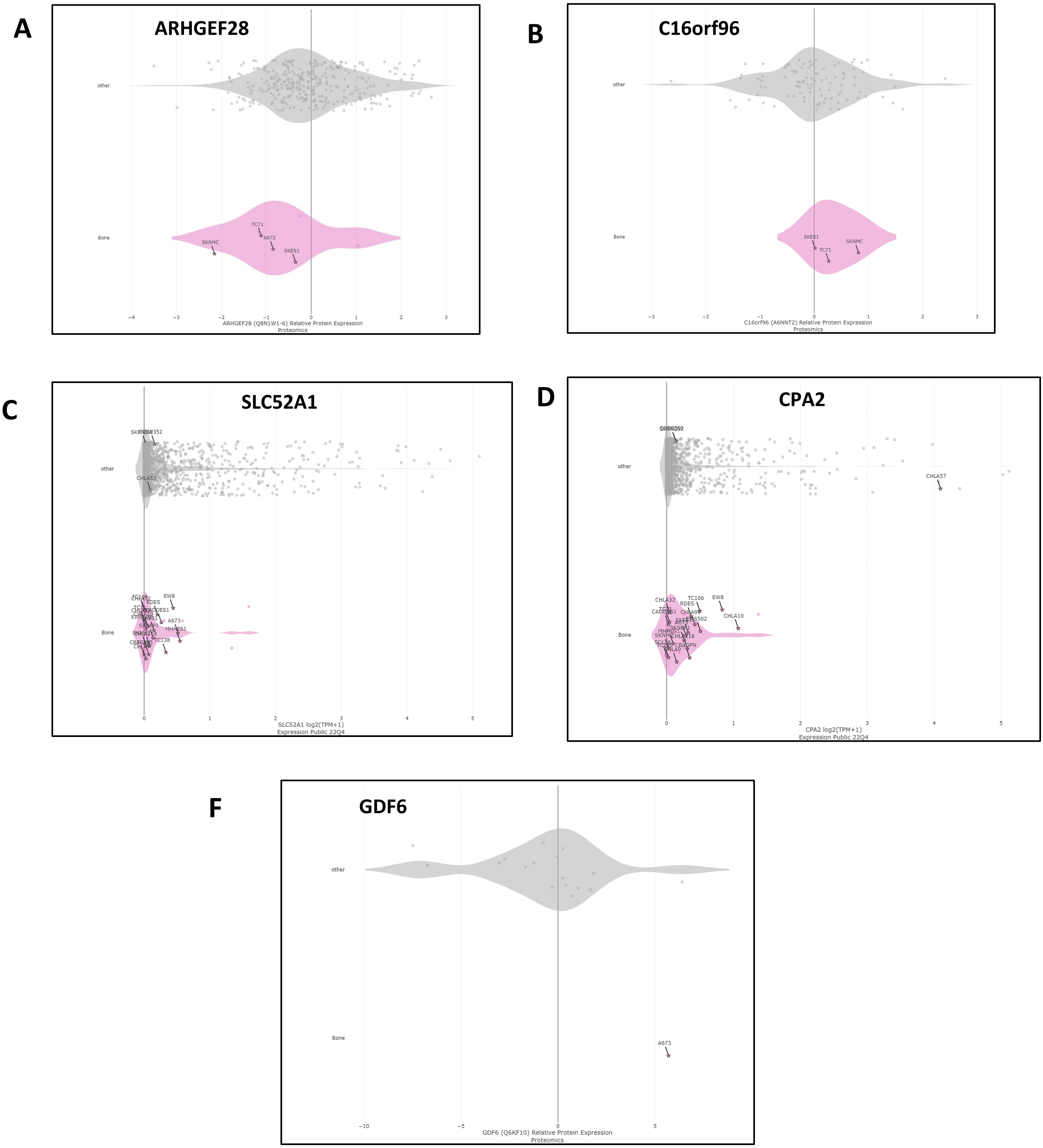

Supplementary Figure 4. RNA expression of identified sEV protein biomarkers in patient samples (BioGPS)

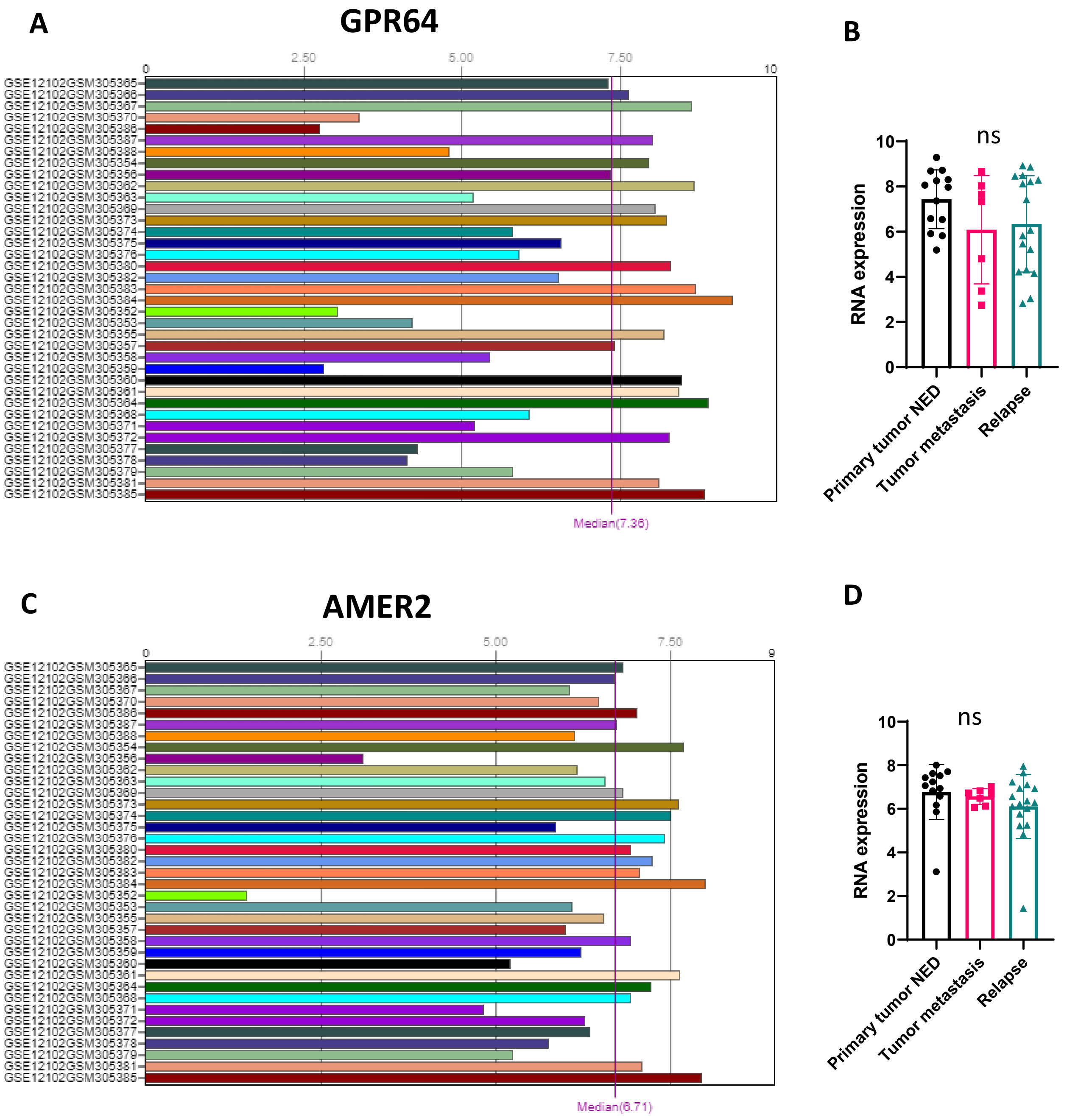

Supplementary Figure 5. Nanoparticle Tracking (NTA) analysis

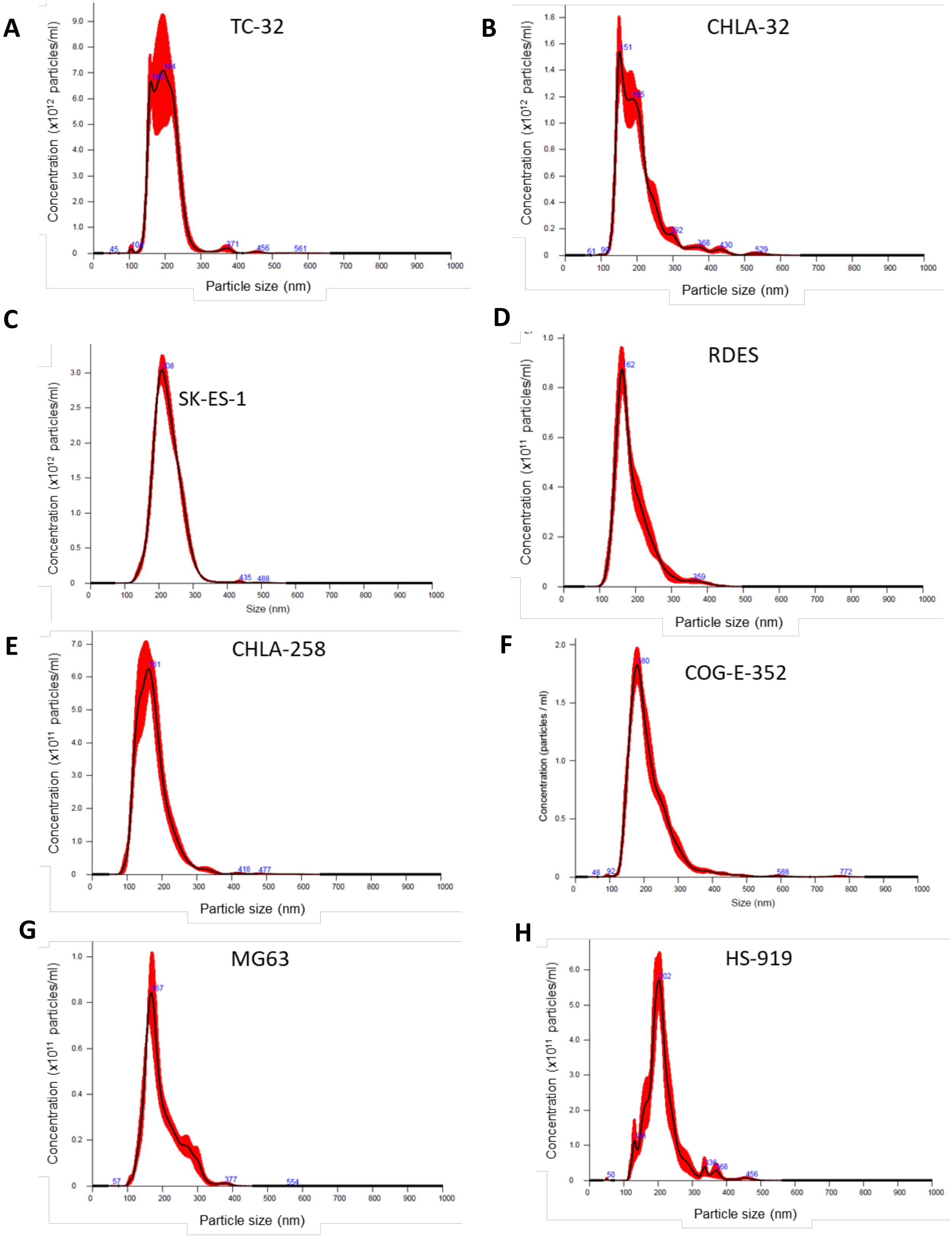

Supplementary Figure 6. Wes protein expression data quantification

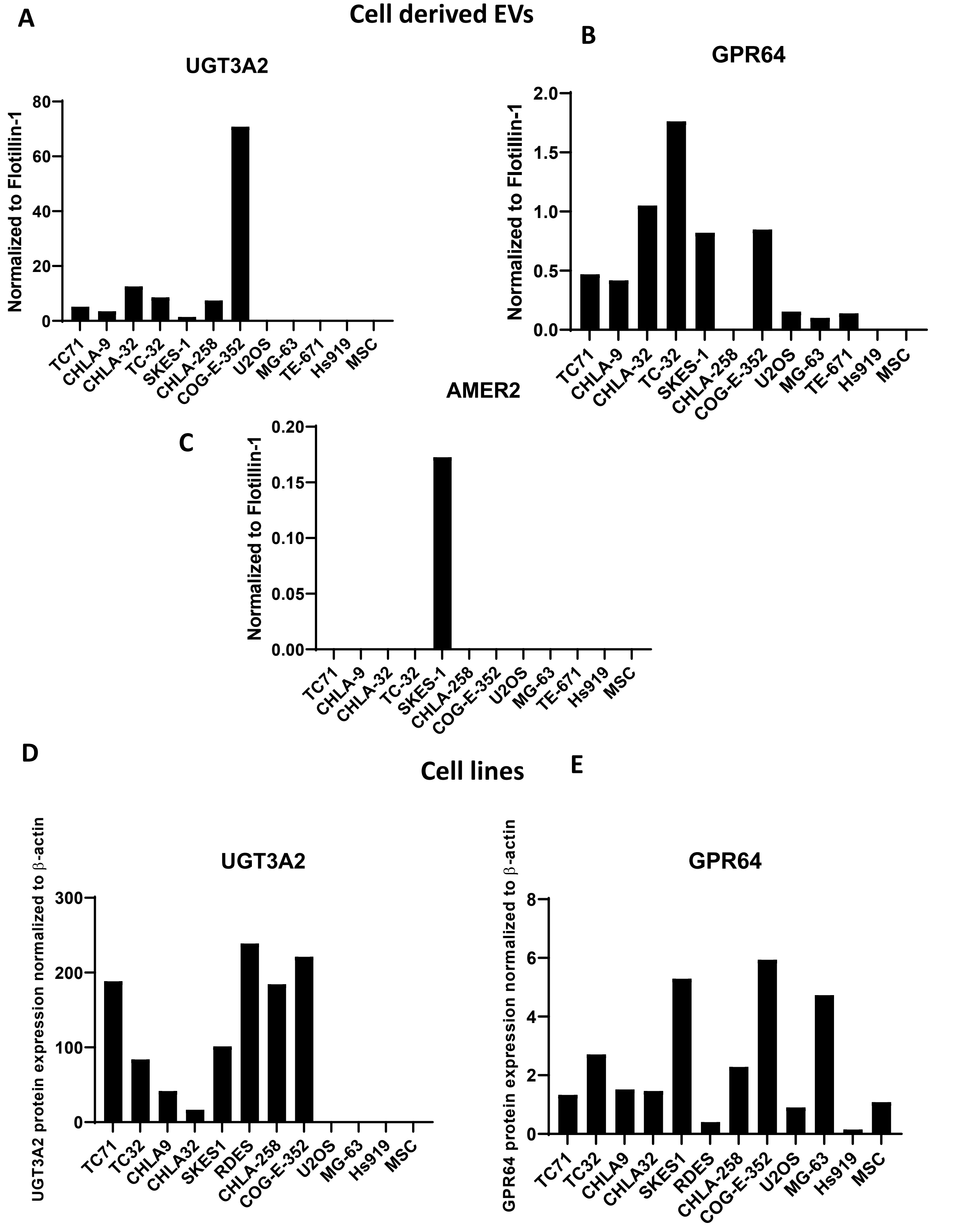

Supplementary Figure 7. UGT3A2 expression in patient sEV samples

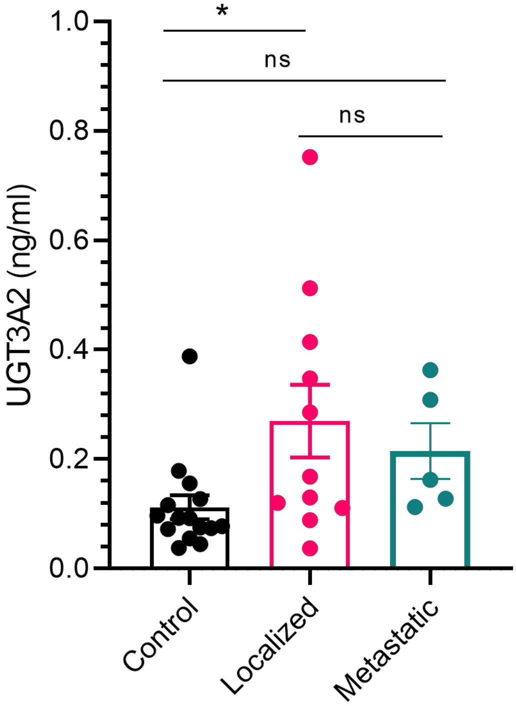

Supplementary Figure 8. Overlap of proteins from previous screen

A

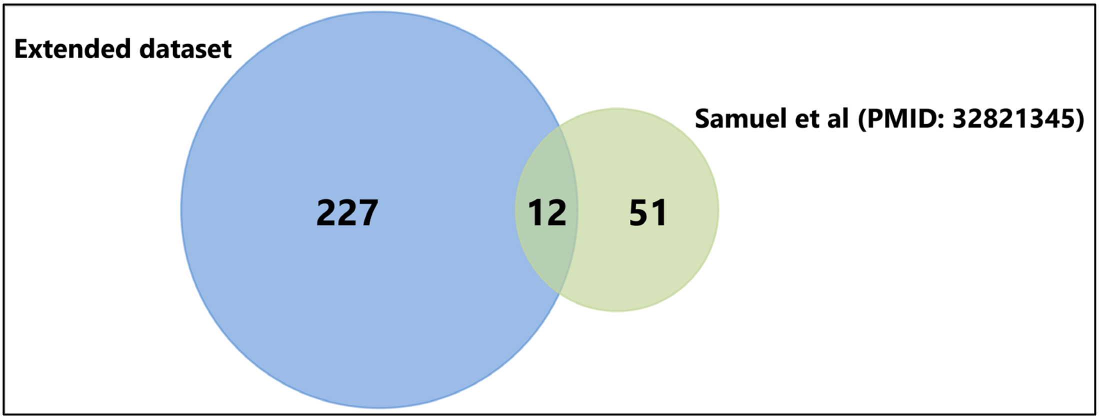

B

Shared protein markers
ENO1
PKM
ENO2
FSCN1
TPI1
HSPD1
TFRC
CAV1
EZR
CD99
HSPA8
STMN1

Supplementary Figure 9. ROC curve based on ELISA for EWS Biomarkers

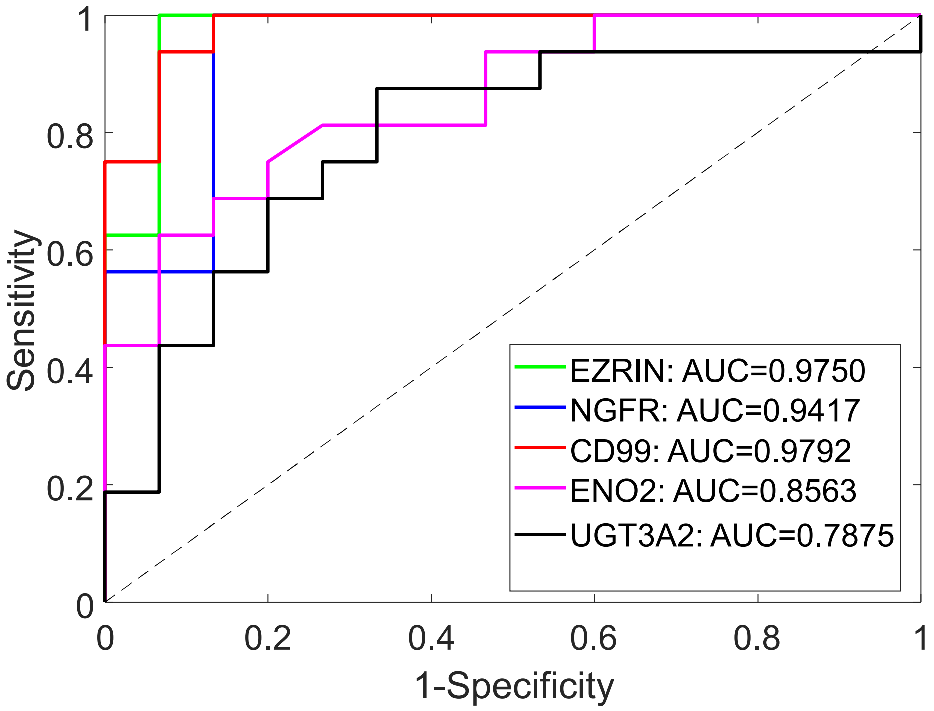

Supplement: Supplementary file 3 [file DataSheet1.PDF]
